# Impact of Removal of Unsatisfactory Eyebrow Permanent Cosmetic Pigmentation on the Quality of Life – WHOQOL‐BREF‐Based Study

**DOI:** 10.1111/jocd.70233

**Published:** 2025-05-22

**Authors:** Weronika Pióro, Bogusław Antoszewski, Anna Kasielska‐Trojan

**Affiliations:** ^1^ Contour Clinic (Private Practice) Wroclaw Poland; ^2^ Plastic, Reconstructive and Aesthetic Surgery Clinic Institute of Surgery, Medical University of Lodz Lodz Poland

**Keywords:** permanent make‐up, quality of life, WHOQOL‐BREF questionnaire

## Abstract

**Objective:**

The appearance of the periorbital area can be improved with eyebrow permanent makeup (PMU); however, the effect is not always acceptable, and the patient may seek to remove it. This study aimed to confirm whether the removal of unsatisfactory eyebrow PMU influences quality of life, and to analyze the factors related to any improvement in quality of life after treatment.

**Methods:**

The study included 75 participants recruited consecutively (mean age: 42.8 ± 10 years); all were female, with Fitzpatrick skin type I‐III, who accomplished PMU removal in one Clinic. During an initial visit, the patients indicated their demographic data, motivation, and reasons for dissatisfaction with PMU in a questionnaire and completed the WHO quality of life brief form (WHOQOL‐BREF). Two treatment modes were implemented: laser treatment or laser plus chemical remover. The WHOQOL was completed a second time at least three months after treatment.

**Results:**

The most frequent cause of dissatisfaction was color (43.8%), followed by color and shape (37%), color intensity/saturation (19.2%), and shape alone (11%). Significant differences in the WHO‐1, WHO‐2, DOM‐1, and DOM‐3 domains of the WHOQOL‐BREF were found between baseline and after treatment.

**Conclusion:**

Removal of unsatisfactory PMU in Caucasian women with Fitzpatrick skin types I–III resulted in significant improvements in overall quality of life and its physical and social domains. The physical aspect shows that the patients consider unwanted PMU as a physical impairment, which, although not being disabling, nevertheless affects body‐related quality of life. In addition, a significant improvement in the psychological domain was also observed after treatment.

## 1 Introduction

Permanent makeup (PMU), also referred to as a *cosmetic tattoo* or *micropigmentation*, is a form of tattoo that is intended to replace or enhance everyday makeup [1] and is frequently used to permanently enhance the appearance of the eyebrows, eyelids, or lips. It involves the application of a natural dye under the surface of the epidermis using a needle [1], hence the name *micropigmentation*. The method serves as permanent makeup, intended to improve the shape and color of different parts of the face and to correct some small defects or asymmetries. Permanent makeup can also serve as an adjunct to counter the negative aesthetic effects of surgical treatment, such as the appearance of scars [2].

Decorative tattooing has been known from antiquity. However, the pioneer of medical dermatography in Europe was the 20th‐century Dutch dermatologist, Eddy Van der Velden, who also designed equipment for dermatography procedures. Since then, dermatography has been successfully performed, i.e., in the cases of pigmented skin lesions, congenital dermatoses, skin defects caused by injuries or surgical interventions; it has also been used in reconstructive surgery, mostly that of the face and breast [3]. The new approach to applying permanent make‐up involves inserting tiny pigment granules under the epidermis, using a needle embedded in a special device. Although the dye gradually fades by about 20% per year, the dye usually lasts from three to 5 years [3] and can thus influence quality of life during this period. However, the somatic and mental functioning of patients undergoing PMU remains unknown.

Although PMU may resemble tattooing in terms of the technique and the pigments used, the two practices have crucial differences in their psychosocial background. The many studies on the motivations and psychosocial characteristics of subjects undergoing tattooing indicate that they generally tend to exhibit risk‐taking tendencies, but have many different aims for body ornamentation [4, 5]. In contrast, the aim of PMU is medical and aesthetic (mimicking makeup) or reconstructive. In this aspect, it seems to be crucial to evaluate how it can affect quality of life, especially in the aspect of purely medical interventions, including its corrections and/or removal.

Due to the increasing popularity of PMU procedures, the number of possible legal complaints has been growing; since 2003, the U.S. Food and Drug Administration (FDA) has received more than 150 formal complaints about PMU complications [6]. This may result from the lack of legal regulations regarding equipment and pigment quality, and the fact that in many countries, no license is required to administer PMU. As any complications and unsatisfactory results are mostly managed by medical professionals, there is a need for an objective evaluation of the outcome with regard to quality of life.

Currently, the impact of cosmetic permanent pigmentation procedures and their correction or removal on patient quality of life (QoL) remains poorly studied. Therefore, the aim of the present study was to identify the main reasons for dissatisfaction with eyebrow PMU and the influence of its correction on patient QoL. The study also analyzed the factors related to improvement in QoL after the applied therapy.

## 2 Materials and Methods

The study involved 73 female participants (mean age 42.8 ± 10 years), whose place of residence was in a city > 100,000 (*n* = 41), a small city (*n* = 21), or a village (*n* = 11). They were recruited consecutively after reporting for eyebrow PMU removal at a private clinic; all underwent the procedure between August 2022 and June 2024. The study included Caucasian women with skin type I‐III Fitzpatrick, in whom permanent make‐up was performed by a professional and who underwent the multistage laser therapy for PMU removal. Selected exclusion criteria for participants included: pregnancy or plans to become pregnant during the duration of the study, diabetes or other diseases affecting the speed of wound healing, previous eyebrow permanent makeup removal procedures, and the presence of excessive tan. All consecutive women who met the criteria and agreed to participate were involved in the study (including taking photographic documentation and filling in the baseline and control life‐quality questionnaire and demographic data form).

During the initial visit, the participants completed a questionnaire on demographics, motivations for PMU removal, and reasons for dissatisfaction with PMU; they also completed the WHO Short Quality of Life Form (WHOQOL‐BREF). Based on the main complaint, one of two modes of treatment was implemented: laser treatment based on a nanosecond Q‐switch Laser Xlase Plus laser (Biotec Italia, Poland) (wavelength of 1064 nm, spot size 4 mm, 3.2 J/cm^2^, 6 Hz and wavelength of 532 nm, spot size 4 mm, 6.4 J/cm^2^, 6 Hz) or laser and chemical removal treatment (Bay‐Bay Pigment Remover Long Time‐Liner, including sodium hydroxide, with alkaline properties at pH 9.0–11.0). The patients were invited to a follow‐up visit at least 3 months after treatment, where they completed the WHOQOL a second time. In addition to the WHOQOL‐BREF (https://www.who.int/tools/whoqol/whoqol‐bref Polish version), the participants completed a questionnaire designed for the study, which included demographic data, information about the permanent make‐up treatment performed, and reasons for dissatisfaction. More specifically, it included the following areas:

- Demographic data (sex, age, education, place of living).
- The PMU (date and course of initial procedure, brand of the pigment, technique, use of local anesthesia, pain during the procedure, complications, healing, overall satisfaction 4 weeks later and satisfaction with the shape or color); it also inquired as to when the participant became dissatisfied with the result and why (color, shape, saturation), when they decided for removal and whether they plan to have more PMU; finally, the participants indicated their degree of satisfaction with PMU removal.
- Questions on general health and medication intake.

The 26‐item WHOQOL‐BREF questionnaire, based on the WHOQL‐100, provides a quality‐of‐life profile based on four domains with appropriate subscales, viz. physical (DOM‐1), psychological (DOM‐2), social relations (DOM‐3), and environment (DOM‐4). Two questions relate to general subjective life quality and an assessment of life satisfaction for the previous 4 weeks (WHO‐1 and WHO‐2). The domain scores reflect the individual perceptions of the quality of life in these areas, with a higher score indicating a better quality of life. The mean values of the items included in each field are used as the final score [7].

The study was approved by the Bioethical Committee of the Medical University of Łódź (RNN/173/22/KE).

### 2.1 Statistical Analysis

The differences in life quality before and after PMU removal were subjected to statistical analysis. In addition, the analysis attempted to identify any factors that correlate with improvement in life quality. The values of individual domains of the WHOQOL‐BREF questionnaire before and after the procedure were compared using the Wilcoxon test. Cohen's d was used as a measure of effect size. The following interpretation was applied: Cohen's *d* = 0.2; 0.5—small effect, *d* = 0.5; 0.8—moderate effect, *d* ≥ 0.8—large effect. To assess whether improvement was more common among participants than worsening, i.e., in more than 50% of cases, the sign test was used. The relationship between the degree of satisfaction with PMU removal and the change in the score for each of the WHOQOL‐BREF domains was investigated using Spearman's rank correlation analysis. In addition, the relationship between the change in the score of each of the analyzed domains and the cause of dissatisfaction with PMU was tested with the Mann–Whitney test. The correlation between the direction of WHOQOL‐BREF domain changes (viz. improvement, no change, or deterioration) and the cause of dissatisfaction with PMU was tested with the chi‐square test.

## 3 Results

### 3.1 Permanent Makeup (PMU) Details

Most participants (*n* = 63, 86.3%) had PMU performed according to the standard scheme, including primary pigmentation and a complementary procedure 4–6 weeks after the first procedure. The rest underwent two pigmentations with an interval of 1 year. Only seven respondents (9.6%) received information about the type and the brand of the pigments used. The techniques used were powder (Ombré Shading) (*n* = 55, 75.3%) and microblading (*n* = 18, 24.7%). While 55 participants (75.3%) received local anesthesia for the procedure, as many as 28 participants (38.4%) recollect the procedure as being painful or very painful. Most respondents observed some skin reactions a few days after the procedure: swelling (*n* = 42, 57.5%), redness (*n* = 67, 91.8%), pain on palpation (*n* = 56, 76.7%), itchiness (*n* = 30, 41.1%) and seroma discharge (*n* = 33, 45.2%). In most (*n* = 44, 60.3%), the symptoms were present for 1 week or just 1–3 days (*n* = 23, 31.5%); however, in six cases (8.2%), they lasted 2–4 weeks.

About 4 weeks after the PMU, 53 patients (72.6%) were satisfied with the result, but 23 (31.5%) claimed the color was too intense or dark, and 15 (20.6%) were not content with the shape (i.e., too wide and/or long). Dissatisfaction with the PMU arose after a year (*n* = 22, 30.1%), 2 years (*n* = 22, 30.1%), or 3 years (*n* = 14, 19.2%); in rarer cases, discontent was noted after a month (*n* = 10, 13.7%) or after a longer period (*n* = 5, 6.8%).

For most participants (*n* = 32, 43.8%), the major cause of dissatisfaction was color, while color and shape were a problem for *n* = 27 (37%), color intensity or saturation for *n* = 14 (19.2%) it and only shape for *n* = 8 (11%). Most decided to undergo PMU removal 2–3 years (*n* = 30, 41.1%) or 4 years (*n* = 20, 27.4%) after its performance; the remainder decided on the procedure earlier. Almost all patients (apart from five) declared that they intended to perform PMU again. Most women indicated on the ten‐point scale that they were very satisfied (8–10 points, *n* = 69, 94.5%) or satisfied (5–7 points, *n* = 3, 4.1%) with PMU removal.

### 3.2 WHOQOL‐BREF

A significant difference in the WHO‐1, WHO‐2, DOM‐1, and DOM‐3 domains of the WHOQOL‐BREF was observed between baseline and after PMU removal, with a small to large effect size. All were higher after removal than before the procedure, with the DOM‐1 values increasing by two points on average (*d* = 1.12) (Table 1).

**TABLE 1:** Comparison of WHOQOL‐BREF results before and after PMU removal.

WHOQOL‐BREF	Mean ± SD	Wilcoxon test	*p*	0.5 SD	ES (Cohen's *d*)
WHO‐1 change	0.315 ± 0.524	4.015	< 0.0001	0.262^a^	0.6 (m)
WHO‐2 change	0.247 ± 0.465	3.621	0.0003	0.232^a^	0.53 (m)
DOM‐1 change	2.00 ± 1.78	6.274	< 0.0001	0.89^a^	1.12 (l)
DOM‐2 change	0.192 ± 1.126	1.842	0.06	0.563	0.17
DOM‐3 change	0.151 ± 0.491	2.395	0.02	0.246	0.31 (s)
DOM‐4 change	0.000 ± 0.601	0.000	1.00	0.3	0.00

Abbreviations: 0.5 SD, a measure of a minimal clinically important difference (MCID); ES, effect size; l – large; m, moderate; s, small.

^a^ Domains presenting MCID.

The percentage of patients who reported improvement in certain domains against the total number of cases registering a change in a certain domain is given in Table 2. The overrepresentation of improvement was significant for DOM‐1, DOM‐2, and DOM‐3. The test was not performed for WHO‐1 and WHO‐2 due to the lack of “deterioration results” for these domains. DOM‐4 was considered in further analysis: no difference between pre‐procedure and post‐procedure outcomes was observed, and the frequency of improvement did not differ significantly from the frequency of deterioration.

**TABLE 2:** Improvement in WHOQOL‐BREF scores after PMU removal.

WHOQOL‐BREF	Improvement (%)	Sign test value	*p*
WHO‐1	100.0		
WHO‐2	100.0		
DOM‐1	95.1	15.987	< 0.0001
DOM‐2	71.0	2.374	0.02
DOM‐3	90.0	3.689	0.0002
DOM‐4	47.8	0	1.00

No correlations were found between satisfaction with the effect of PMU removal and the change in the WHOQOL‐BREF (Table 3) within each domain, in none of the three groups: in the group where the domain score improved, in a group where there was no change in the domain score, and in a group where the domain score deteriorated. The cause of dissatisfaction with PMU was also compared with the changes in the WHOQOL‐BREF results. The only significant relationship observed between dissatisfaction with eyebrow and the change in the WHO‐1 score, i.e., those who indicated that they were dissatisfied with the shape of PMU showed a significantly higher mean change in the WHO‐1 score (0.514 vs. 0.135) (see Table 4).

**TABLE 3:** Relationship between satisfaction with the outcome of PMU removal and change in WHOQOL‐BREF score.

WHOQOL‐BREF	Correlation – *R* ^2^	*T*‐test for *R*‐factor	*p*
WHO‐1 delta vs. Satisfaction	0.142	1.145	0.26
WHO‐2 delta vs. Satisfaction	0.020	0.162	0.87
DOM‐1 delta vs. Satisfaction	−0.076	−0.609	0.54
DOM‐2 delta vs. Satisfaction	0.088	0.710	0.48
DOM‐3 delta vs. Satisfaction	−0.162	−1.317	0.19

**TABLE 4:** *p*‐values for the relationship between the cause of dissatisfaction with the PMU and the change in WHOQOL‐BREF domain scores after its removal (Mann–Whitney test).

WHOQOL‐BREF	Color	Shape	Saturation	Other
WHO‐1	0.56	0.02	0.70	0.66
WHO‐2	0.24	0.26	0.96	0.59
DOM‐1	0.24	0.20	0.35	0.15
DOM‐2	0.55	0.52	0.09	0.41
DOM‐3	0.23	0.39	0.23	0.41

Other significant relationships were noted for the following: 1—a change in WHO‐1 and eyebrow shape, i.e., improvement in WHO‐1 was significantly more common among participants who complained about the shape of the eyebrows (45.7% vs. 13.5%); 2—a change in DOM‐2 and PMU saturation, i.e., improvement in DOM‐2 was significantly more common in those who indicated dissatisfaction with pigment saturation (57.1% vs. 24.1%) (Table 5).

**TABLE 5:** *P*‐values of the relationship between the cause of dissatisfaction with the PMU and the direction of WHOQOL‐BREF domains score change (viz., improvement, no change, deterioration) after its removal (Chi‐square test).

Specification	Color	Shape	Saturation	Other
WHO‐1	0.48	0.003	0.55	0.51
WHO‐2	0.10	0.01	0.83	0.42
DOM‐1	0.31	0.73	0.69	0.43
DOM‐2	0.76	0.37	0.04	0.77
DOM‐3	0.12	0.33	0.12	0.26

**FIGURE 1:**
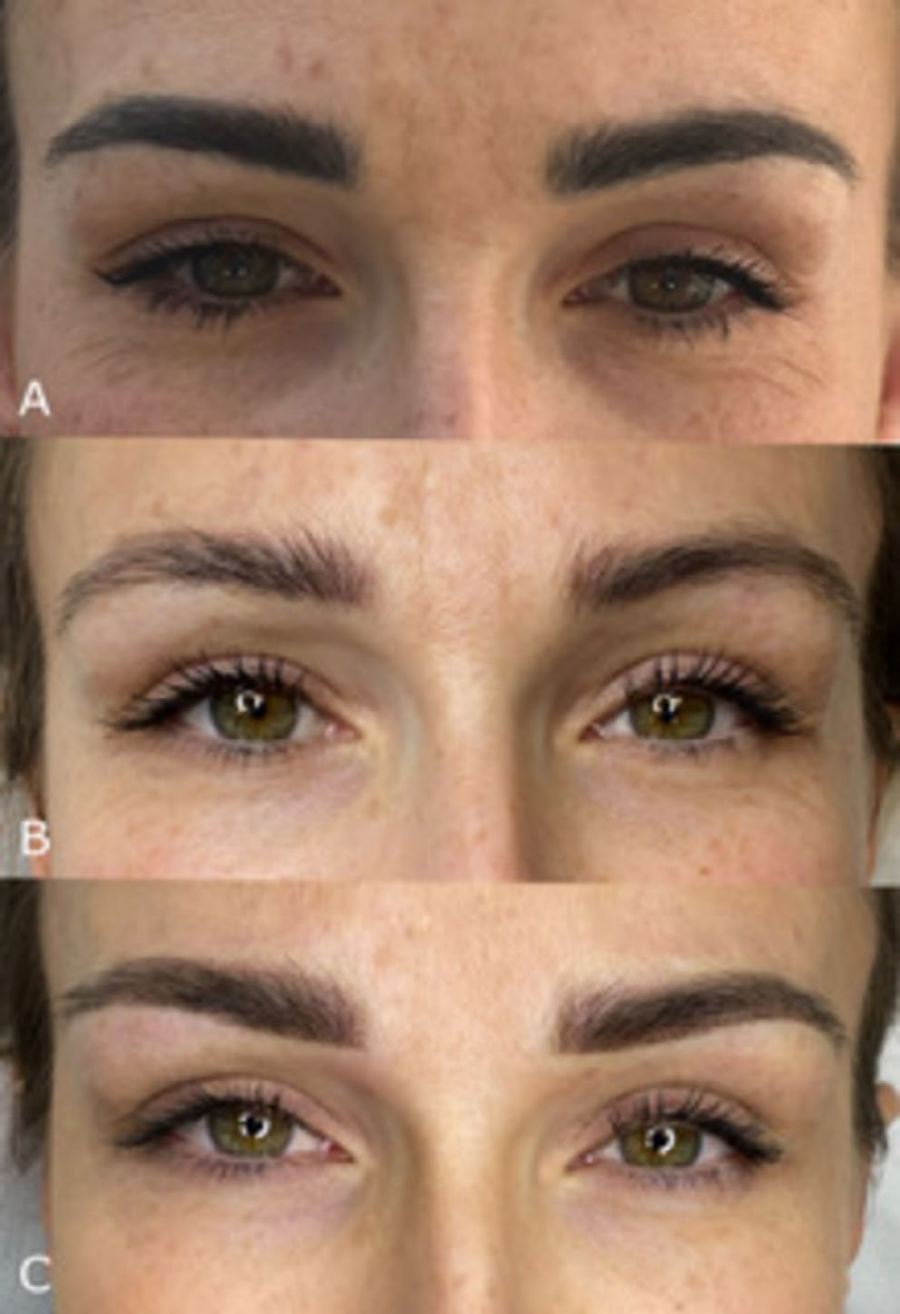
A patient dissatisfied with PMU color and saturation (A—before removal, B—after PMU removal, C—after performing new PMU).

**FIGURE 2:**
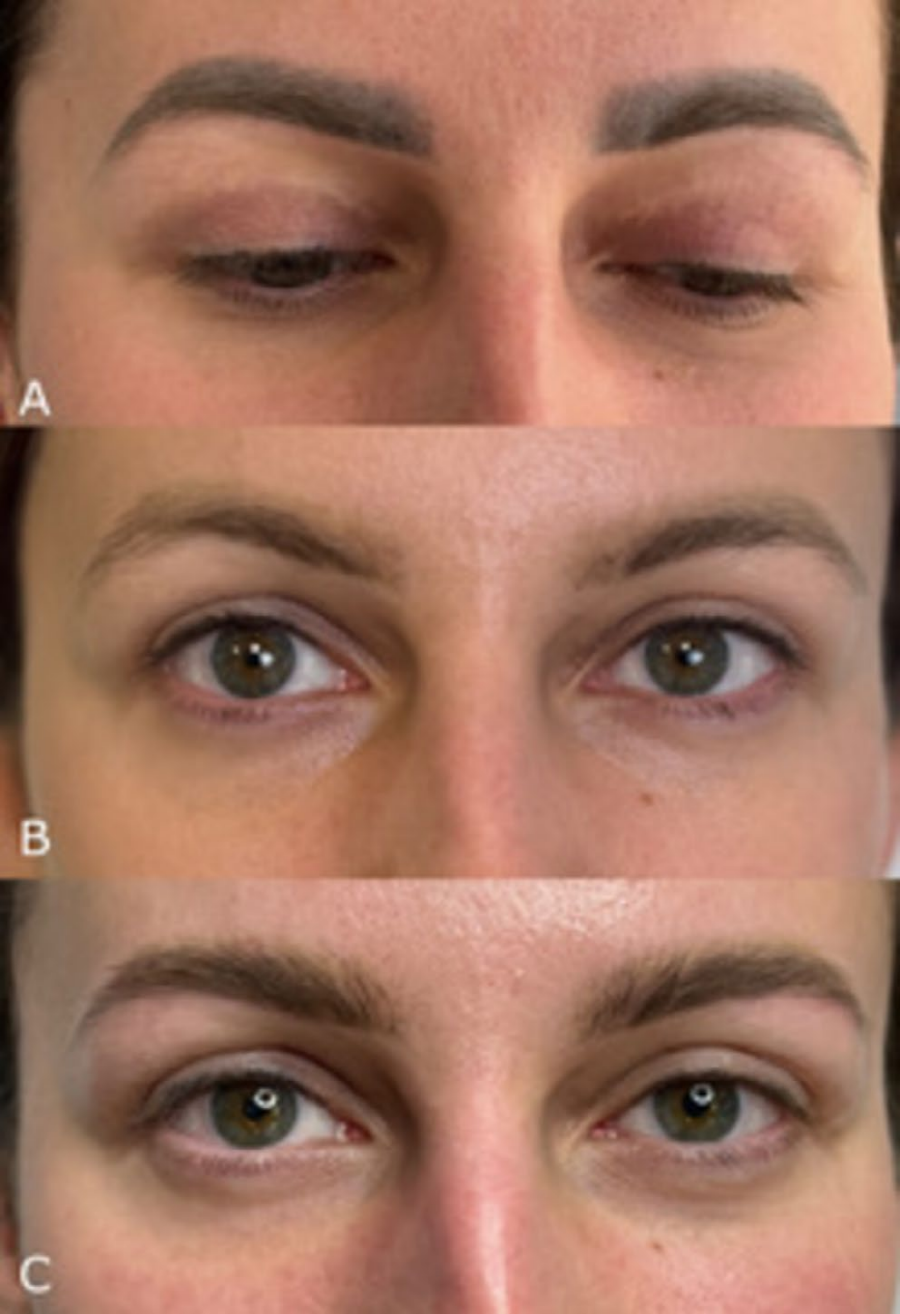
A patient dissatisfied with PMU color and shape (A—before removal, B—after PMU removal, C—after performing new PMU).

Dissatisfaction with the color and shape of PMU after laser removal, and the rating of the new, satisfying PMU are given in Figures 1 and 2.

## 4 Discussion

The present study examines the influence of unsatisfactory PMU removal on quality of life (QoL). It also analyzes the reasons for dissatisfaction with PMU and identifies the variables that correlate with improvement in QoL. It was found that the physical and social relations domains improved significantly after removing unsatisfactory PMU, as did WHO‐1 (general evaluation of one's own quality of life) and WHO‐2 (satisfaction with general health). Also, it appeared that the improvement in general life quality (WHO‐1) observed after PMU removal was associated with dissatisfaction with the shape of the PMU.

Quality of life consists of the patient's functioning in the physical, emotional, and social spheres. In clinical practice, QoL should be assessed both subjectively and objectively [8]. A study of the Polish WHOQOL‐BREF based on a group of 908 respondents found that the psychological domain contributed most to general health satisfaction, considered as a dependent variable, followed by the physical and environmental domains; in contrast, the physical domain appeared to best differentiate between unhealthy and healthy patients, followed by the psychological and social domains [9].

It has also been found that among women, eyebrow movement is used to convey emotions through non‐verbal communication, and that it is a clear feature of the human face and provides gender identification; as such, the brows appearance influences quality of life [10].

The face is also believed to play an important role in interpersonal interactions, with the orbitofrontal area being particularly important for recognition and attractiveness. Among the various studies on the beauty of the orbitofrontal region, some indicate that attractiveness is determined by aspects of evolutionary psychology, such as symmetry, dimorphism, and facial expression. Their findings indicate the ascending interangular axis and the eyebrow axis of the brow to be the main criteria of biological attractiveness, and thus a higher quality of life [11]. This observation was confirmed by Sclafani and Jung, who found the aesthetic position of the middle eyebrows to be relatively low; the authors propose that the top of the eyebrows should be just medial from the side corner of the eye to avoid creating an unnatural look for the eyebrows [12]. In addition, eyebrow restoration has become a part of the anti‐aging procedures of the face. With the rise of PMU popularity and the growth of the ‘beauty industry’, dermatologists are involved in recognizing and dealing with the potential complications and consequences of these procedures to facilitate proper patients' care [13, 14] Therefore, in the future, it is necessary to clarify and develop public interest in cosmetic procedures in relation to the medical aspects of treatments (i.e., guide complications management, develop and implement standards regarding devices and chemicals used for the procedures) [15]. Analysis of the types of dyes used in tattooing and PMU points to the health risks and side effects associated with their use. There are still no standardized methods for analyzing pigments and inks for assessing their safety. Although the concept of tattooing has been around for millennia, effective legislation has yet to be implemented. Currently, in Europe, these issues are covered by the general REACH regulation (ResAP Resolution, 2008; EU Regulation 2020/2081, 2020) on product safety, with the new amendment (from 4 January 2022) concerning limits on the concentration of certain substances used in tattoo inks and PMU pigments. However, these laws do not sufficiently protect either the consumer or the industry itself [4].

Our results indicate that although color was the most frequent cause of dissatisfaction with PMU, dissatisfaction with shape correlated with higher improvement in general life quality after its PMU removal. Park et al. [16] also reported various aspects of dissatisfaction with PMU of the scalp and noticed significant improvement in life quality after its removal. He also pointed out that satisfactory results can be achieved through appropriate corrections, such as removing pigmentation and redoing it. Drost et al. [17] aimed to analyze the effect of dermatography on the subjective perception of the appearance of scars and skin grafts and the quality of life in head and neck patients. They concluded that the technique is a useful adjuvant procedure to improve the subjective perception of scar and skin graft appearance and the quality of life in patients after tumor resection and reconstruction. The study also highlighted the role of life quality studies in analyzing the role of PMU in medical therapies.

However, PMU removal can result in infectious, allergic, and inflammatory complications, as well as undesired changes in the appearance of the PMU, mostly discoloration, which may be considered a complication. Dermatologists should be aware of the potential complications and be able to treat such unwanted effects [6]. Our analysis showed that for most of the participants, the most common individual cause of dissatisfaction was color alone, followed by both color and shape; shape alone was rather a rare cause of dissatisfaction.

Dissatisfaction most commonly appeared about 1 or 2 years after the procedure, which would coincide with the appearance of discoloration. This highlights the need for stable and good‐quality pigments. This is particularly important when PMU is used for medical reasons, e.g., as a complementary procedure to improve color matching in the head and neck area. Dermatography is regarded as an effective complementary procedure, improving the subjective perception of the appearance of scars and skin grafting, as well as QoL, in patients with head and neck diseases [13]. Our findings also indicate that, as correction of undesired PMU significantly improves quality of life, it should be considered a medical procedure. The clinical effect of this influence is, however, difficult to evaluate. The minimal clinically important difference (MCID) is an important concept in life quality studies, trying to interpret quality of life measures in relation to other patient‐reported outcomes, being a bridge between statistics and clinical meaning. In the context of WHOQOL‐BREF, it can be seen as questionnaire dimension score change recognized clinically by the patient [18]. Our study showed that satisfaction from the therapy of PMU removal was associated with the change (increase) in DOM‐1 of an average of 2 units and less than 1 unit in DOM‐2 and DOM‐3. However, further studies should be carried out to verify this observation, as in our study, most patients were satisfied with the treatment. Moreover, the interpretation of MCID depends on the patient population from which it is derived, so the determination of a threshold for the MCID of WHOQOL‐BREF in patients undergoing PMU removal requires evaluation of this specific group of patients. There are different MCID calculation methods, and one of the distribution‐based estimates of the MCIDs for the different domains of the WHOQOL‐BREF is 0.5 times the standard deviation (SD) of the Δ score (change). The 0.5 SD benchmark of an outcome measure means that patients improving more than 0.5 of the outcome score's SD have reached a minimal clinically important difference [19]. Our results (see Table 1) showed that for the three domains for which the effect size was moderate or large (WHO‐1, WHO‐2, DOM‐1), the observed changes were clinically important.

The study has a few limitations. First, the studied population may seem small, especially considering the popularity of PMU; however, the study only included patients who were truly dissatisfied with the results and who underwent pigmentation removal. In addition to excluding the effect of dissatisfaction with incomplete pigment removal, all participants had to successfully complete the multistage laser therapy. For the same reason, the study did not analyze the impact of the different therapies (laser vs. laser and chemical remover) on QoL. Further, all participants were Caucasian, so the results can apply only to this population of women with rather fair skin (Fitzpatrick 1‐III), which could also increase the visibility of color‐related aspects of pigmentation that impact quality of life.

Nevertheless, this study is the first to analyze the impact of unsatisfactory eyebrow PMU on quality of life. It also indicates that correction of the undesired color and shape of the eyebrows improves the quality of life of the patient and affects their psycho‐social functioning. Due to the widespread nature of cosmetic procedures involving PMU, there is a need for a better understanding of their impact on the social function of patients. Our results suggest that all practitioners involved in permanent modifications, especially within the facial area, should be licensed professionals, as undesired effects may have a negative influence on the quality of life and functioning of the patient. It is also recommended that, in the event a cosmetic procedure has an undesired result, a trained medical specialist should be available due to the potential risks of its correction. Laser therapy is considered a safe and effective method of PMU removal; however, its efficacy depends on the color of the pigment. The laser wavelength that is effective for the removal of black, gray, and blue is 1064 nm, while red and orange pigment is reached by waves of the length of 532 nm. But this wavelength is not recommended for patients with darker skin due to the risk of skin discoloration or depigmentation [20]. Further complications of the therapy may result from the change in the physicochemical properties of the pigment particles absorbed by the macrophages and may include severe allergic reactions [21]. Chemical removers' complications have not been studied, but may be similar to chemical peels' side effects.

## 5 Conclusion

Removal of unsatisfactory PMU in Caucasian women with Fitzpatrick skin types I–III improved general quality of life and its physical and social domains, among the tested patients. The improvement in the physical domain shows that affected women consider unwanted PMU as a physical impairment, which, although not disabling, affects their body‐related life quality. Also, improvement was observed significantly more frequently for the psychological and social domains. Undesired shape correction and/or removal correlated with the improvement of the general quality of life. However, due to the scarce literature, further research on the influence of PMU and its removal on life quality is necessary, especially including international samples. Our findings support the consideration of PMU removal as a medically relevant aesthetic procedure with an impact on quality of life, which emphasizes the need for PMU practices legal regulations.

## Author Contributions

All authors contributed to the study conception and design. Material preparation, data collection, and analysis were performed by Weronika Pióro and Anna Kasielska‐Trojan. The first draft of the manuscript was written by Weronika Pióro, and all authors commented on previous versions of the manuscript. All authors read and approved the final manuscript.

## Ethics Statement

This study was performed in line with the principles of the Declaration of Helsinki. Approval was granted by the Bioethical Committee of the Medical University of Łódź (RNN/173/22/KE).

## Consent

Informed consent was obtained from all individual participants included in the study. The authors affirm that human research participants provided informed consent for the publication of the images in Figures 1 and 2.

## Conflicts of Interest

The authors declare no conflicts of interest.

## Data Availability

The data that support the findings of this study are available from the corresponding author upon reasonable request.
